# Rheumatism and Gout

**Published:** 1904-11-05

**Authors:** 


### RHEUMATISM AND GOUT.

**Acute Rheumatism.**—Poynton [1] gives an interesting account of recent advances and shows how, step by step, the difficulties are being cleared up. Both he and Shaw produced a non-suppurative iritis in rabbits by injecting the diplococcus from rheumatic fever. Shaw, too, shows that the organisms isolated by Wasserman, by Poynton and Paine, and by Ainley, Walker, and Beatson are one and the same. Choreiform movements have been produced by Beattie's injection of this diplococcus. One of its peculiarities is the large amount of formic acid it produces, far in excess of that given off by streptococci. Appreciable amounts are found in the urine of rheumatic patients and are lessened when salicylates are given. Menzer, in Germany, impressed by the frequent co-existence of tonsillitis and rheumatism, thinks the latter is due to a streptococcal infection and devised a streptococcal serum to cure it, but the results do not appear very satisfactory. Another theory that rheumatism is due to an attenuated pyæmia has been investigated by Poynton and Shaw. Taking the staphylococcus aureus as a common cause of pyæmia they attenuated it by growing it on a special medium and then injected it into animals, but the result was always either septicæmia or no effect, when they injected the staphylococcus together with the diplococcus, the result again was of a pyæmic nature, and they conclude that the staphylococcus is not a cause of rheumatism either alone or when mixed with other organisms. Poynton [2] reports also the case of a girl who suffered from chorea and affections of the heart and joints. A year afterwards she died with symptoms of malignant endocarditis, and small vegetations were found on the mitral valve—cultures yielded a diplococcus of the type previously referred to, which produced when injected into animals arthritis and cardiac lesions. He looks on this case as confirming the infective nature of rheumatic fever. Though he certainly shows here that a special diplococcus is productive of a form of endocarditis which may be fatal and is often met with in cases held to be rheumatic fever, it has been pointed out that this patient died from heart disease, and it is not certain that organisms found at death were the cause of the rheumatic symptoms months or years before. Hence it is not in strictness a proof that rheumatism is even at times due to this organism. So, too, the presence of the coccus in rheumatic nodules is no proof of their being caused by it. Still the large number of cases in which the injection of the diplococcus has been followed by joint and other apparently rheumatic lesions in animals is a fact which cannot be put aside. As Poynton says, the organism is found in all cases where it could be expected; it is found in the local lesions, produces similar lesions in animals, and is itself reproduced in them. Certain other facts are also clear, namely the existence of rheumatic bronchopneumonia, pleurisy and peritonitis, and of renal rheumatism, while it is possible to produce heart lesions at will by injections of the diplococcus.

**Epigastric Pain** in acute rheumatism in children is not uncommon. S. Vere Pearson[3] finds that it usually starts from one or both sides, occurs in paroxysms after some exertion several times a day, is not connected with food or gastric troubles, but is accompanied by or followed after some days by symptoms of ordinary rheumatism. Ralph Stockman[4] analyses the symptoms of muscular rheumatism which he thinks is not a stage of the acute disease though it may be due to an attenuated form of the same organism. The acute type often follows a chill and slight sore throat. There is little fever, but there are pains in the muscles and bones, with tenderness and some diffused swelling or doughiness over them. All of these symptoms pass off in a few days. In chronic cases fibrous indurations exist, and attacks of pain for weeks together follow any exertion or exposure to cold and damp. The indurations may press upon nerves and lead to severe neuralgia. They can be felt by careful manipulation as fibrous nodules or cords, or softer ill defined masses in the muscles and along their insertions and aponeuroses. Salines and diaphoretics with massage are of use in the acute cases; the salicylates are not so efficacious as in rheumatic fever, but greatly relieve pain when given internally or by inunction. Phenacetin is often useful. In chronic cases local massage must be continued for weeks; the indurations at first become larger and more painful, but by daily gentle rubbing they decrease and become hard and callous, and finally disappear. The faradic current may be applied daily over the nodules as an aid to, or in place of, the massage. Injections of 5 drops of a 1 per cent. solution of chromic acid may be made into sharply defined nodules. Internal remedies or a special diet give little relief, but intestinal fermentation should be prevented. Mendel[5] has employed intravenous injections of salicylate of soda and caffeine in various forms of rheumatism, especially in chronic joint cases with little pyrexia, or in acute rheumatism when the drug given by the mouth has lost its effect or fails to relieve certain joints. A formula used by him is sodii salicylatis 8 75, caffein 1·25, aquæ destillatæ ad 50. Two grammes of this solution were, in the case of adults, injected into a large vein of the arm. The vein should be previously distended by an elastic band, and the site rubbed with ether. A solution of salicylate of soda alone is not well borne as an intravenous injection. Carey Coombs[6] points out certain clinical features which are common to all rheumatic affections, just as there is a common histological picture presented by rheumatic lesions in various parts of the body; that is a central necrosis surrounded by proliferation and fibrosis, and that again by a zone of hyperæmia. So, too, on the clinical side there are transient cases in which the invasion is quickly defeated, and malignant ones which are rapidly fatal. Between these two there are protracted mild cases which relapse and recur, and chronic cases which end with a malignant outburst. Thus the arthritis, the endocarditis, or chorea may all occur as transient, malignant, or protracted attacks, the disease in the last case lying latent for a while, but breaking out from time to time to the final destruction of the tissues. He considers that many cases of rheumatoid arthritis are due to these smouldering invasions of the rheumatic poison.

**Arthritis Deformans.**—Macrae ([7]) holds that rheumatoid arthritis is distinct from true rheumatism and may be divided into the following classes:—(*a*) Heberden's nodes, (*b*) monarticular forms, (*c*) spondylitis, (*d*) polyarticular forms, either acute or slowly progressive. Poynton, on the other hand, thinks that many forms of rheumatoid arthritis are true rheumatism. Erving investigated the blood and found that the red cells were above normal, and the hæmoglobin high, which is contrary to the general belief. Hale White ([8]) classifies these forms of arthritis as (*a*) the osteoarthritis of the aged, (*b*) the polyarthritis of middle-aged women, with much bony outgrowth and eburnation of the cartilages, (*c*) the type seen in young women beginning in the proximal interphalangeal joints and wrists, and sometimes affecting every joint in the body in a few weeks. There is in this last no affection of bone or cartilage, but a thickening of the synovial membranes. However, the Roentgen rays show in some cases a remarkable transparency of the ends of the bones, probably due to a deficiency of the bone salts. This may be noticed on either side of the metacarpophalangeal joints, at the ends of the phalanges, and also at the shoulders, ankles, and knees. In this type of case, too, there is evening pyrexia, rapid pulse, sweating of the hands and feet, pigmentation, and remarkable wasting of the muscles. The writer is inclined to think this acute form is a distinct disease. He notices too the probability that some cases regarded as rheumatoid are tubercular. This is especially true of the peculiar type described by Still in children. Others are certainly due to the slow absorption of septic material from a chronic abscess, such as caries of the jaw, and of this class pulmonary osteo-arthropathy is probably an instance.

[1] Pract., June. [2] Brit Med. Jour, May 14. [3] Brit. Med. Jour., May 14. [4] Brit. Med. Jour., Feb. and March. [5] Scottish Med. and Surg. Jour., June. [6] Lancet, Feb. 27. [7] Pract., June. [8] Bristol Med. Chir. Jour., June.

